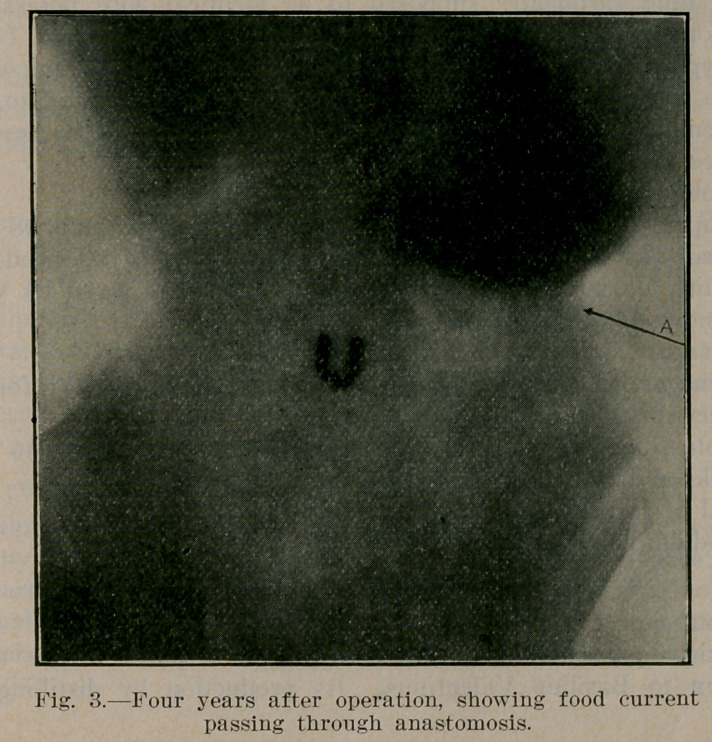# Congenital Hypertrophic Stenosis of Pylorus

**Published:** 1913-06

**Authors:** 


### ABSTRACTS.

---

CONGENITAL HYPERTROPHIC STENOSIS OF PYLORUS Dr James B. Eagleson, Seattle, Wash., *Northwest Medicine*, April, 1913, reviews the history and states that the mortality under medical treatment is 80-90 per cent. While the first description of the disease is usually attributed to Hirschsprung, twenty-five years ago, Osler found a clear account by Hezekiah Beardsley, with description of necropsy, in Cases and Observations by the Medical Society of New Haven County, Conn., 1787. Fredet, in September, 1910, tabulated the surgical procedures and results, as follows:

Pylorectomies 1, mortality, 100 per cent.
Loreta divulsions 39, 15 deaths, 24 recoveries.
Pyloroplasties 26, 11 deaths, 15 recoveries.
Modified pyloroplasties 17, 3 deaths, 14 recoveries.

Fig. 1.—Five years after operation, showing food current passing through pylorus.

Gastro-enterostomies:
Not named 17, 10 deaths, 7 recoveries.
Anterior 20, 13 deaths, 7 recoveries.
Posterior 49, 21 deaths, 28 recoveries.

Of these various operations pylorectomy was early abandoned as useless in a benign tumor. The divulsion of Loreta gave a high percentage of recoveries but many relapsed and had to have a second operation. Several of the deaths were from hemorrhage and rupture of the duodenum. Pyloroplasty is a very difficult operation in pyloric hypertrophy, and, while it has given fairly good results in the hands of European, has never been in favor with American surgeons.

Fig. 2.—Four years and four months after operation, showing food current passing through anastomosis.

Fig. 3.—Four years after operation, showing food current passing through anastomosis.

The technic of posterior gastro-enterostomy has now been so carefully perfected and requires practically no longer time than any of the other operations, that it has become the operation of choice.

Dr. Eagleson reports three cases, all in male infants, treated by posterior gastro-enterostomy, successfully. Each of the illustrations shows the after result in these cases, respectively. The illustrations are reproduced through the courtesy of the author and the Editor of *Northwest Medicine*.

The Evolution of the Chemical Materia Medica. The following notes were abstracted for the *Critic and Guide* by Martin I. Wilbert, Philadelphia, Pa., from a list published in the *Pharmaceutische Post:*

SUBSTANCES KNOWN TO THE ANCIENTS.

Sulphur; this substance is mentioned in the oldest scientific works.

Ammonium chloride; sal ammoniac was known to Herodotus, and it is said to have been discovered in the neighborhood of a temple dedicated to Jupiter Ammon, in Libya.

Realgar and sulphide of antimony.

Potassium carbonate; known to Dioscorides.

Sodium carbonate; supposed to be identical with potassium carbonate. Identified by Duhamel in 1736, and Marggraf, 1759.

Gypsum, lead carbonate, lead oxide, iron, ferrous sulphate, alum, zinc ores, zinc oxide, known as cadmia or pompholix, to the alchemists as "Lana philosophica," or, on account of its resemblance to snowflakes, "Nix alba."

Gold, silver, copper, as "Aes cypricum."

Cupric sulphate, known to the Greeks as chalcanthum, to the Romans as atramentum sutorium. More closely described by Basilius Valentinus. Directions for making were given by Van Helmont, 1644, and Glauber, 1648.

Mercury; known to Aristotle.

Vinegar, lead plaster, soap; the earliest descriptions ar found in the works of Pliny.

Potassium bitartrate; in the crude state this was known to the Greeks and Romans, to the latter as "Fæx vini."

Oil of turpentine.

Sodium chloride.

SUBSTANCES INTRODUCED BY THE ARABIANS.

Potassium nitrate; called by Geber "sal petræ."

Acid, hydrochloric, in Aqua Regia. The purer article was known to Basilius Valentinus. Its production by distilling a mixture of sodium chloride and sulphuric acid was described by Glauber, hence its name, "Spiritus fumans Glauberi."

Acid, nitric; known to Geber.

Arsenous acid, "white arsenic, "known to Geber. More reliable data were not obtainable until the middle of the eleventh century.

Mercuric chloride; known to Geber, Rhazes, and also to Avicenna.

Mercuric oxide; known to Geber.

Silver nitrate; known to Geber, but was introduced into medicine by Angelus Sala during the seventeenth century, when it was known as "Magisterium Argenti," "Crystalli Dianæ."

Alcohol, dilute; stronger alcohol was first produced by Raymundus Lullus in the thirteenth century, who introduced it into the materia medica as "ultima consilatio corporis humani."

Lead acetate; although this was known to Geber, it was not introduced or used as a medicine until 1760, when Goulard produced what was later known as "Aqua vegetomineralis Goulardi."

Camphor; this was first brought to Europe about the middle of the sixth century.

Aqua ammoniae; was known to Beber, as was also a more or less pure caustic potash.

SUBSTANCES KNOWN TO, OR INTRODUCED BY, RAYMUNDUS LULLUS IN THE THIRTEENTH CENTURY.

Alcohol; stronger.

Ammonium carbonate; produced from urine.

White precipitate.

THE FIFTEENTH CENTURY CONTRIBUTED:

Potassium sulphate; this may have been known to Isaac Hollandus in the fourteenth century. Described by Oswald Croll, about 1608, as "Specificum purgans Paracelsi."

Sulphuric acid; the first known accurate description of this substance is attributed to Basilius Valentinus.

Zinc sulphate, "white vitrol"; known to Basilius Valentinus.

Ferric chloride, lead acetate, and the spirit of nitrous ether were all known to Basilius Valentinus.

THE SIXTEENTH AND SEVENTEENTH CENTURIES GAVE US:

Sulphuric ether; discovered by Valerius Cordus about 1540, and by him described as "Oleum vitrioli dulce"; this preparation appears to have been forgotten until rediscovered by Frobenius, a London apothecary, about 1730.

Mercurous chloride; known in Europe in the sixteenth century.

Oil of anise, oil of cloves; known to Valerius Cordus.

Benzoic acid; known about 1608.

Antimony and potassium tartrate; produced by Adriano Van Mynsicht, 1631.

Zinc chloride; mentioned by Glauber, 1648, as "Oleum lapidis caliminaris."

Sodium sulphate; known to Glauber, 1658.

Potassium permanganate; Glauber, in 1659, noted the peculiar color that was produced on fusing potassium nitrate with manganese dioxide. The composition of potassium permanganate was first described by Mitscherlich in 1830. The name "Chamælon minerale" was given to it by Scheele.

Ammonium acetate; was introduced by R. Minderer during the seventeenth century.

Phosphorus; discovered by Brand, in urine, about 1669. More carefully studied by Gahn, 1769, and by Scheele, 1771, who demonstrated its presence in bones.

Sodium borate; probably known at an earlier period, was reintroduced during the seventeenth century by the Venetians.

Potassium and sodium tartrate; introduced about 1682 by an apothecary, P. Seignette, of Rochelle, France.

Magnesium sulphate; discovered 1694, by Nehemiah Grew, in the water of a mineral spring at Epsom, England.

DURING THE EIGHTEENTH CENTURY THERE WERE INTRODUCED:

Magnesium carbonate; introduced as a secret remedy; "Magnesia alba," about the beginning of the eighteenth century. The method of preparing was described by Valentini, 1707, and Slevogt, 1709. The composition of "Magnesia alba" was demonstrated by Black, 1756.

Boric acid; produced by Homberg in 1702 by the decomposition of borax. Known as "Sal sedativum Hombergi."

Phosphoric acid; produced in 1746 by Marggraf.

Magnesium oxide; in 1755 by Black.

Ether, acetic; first produced by Lauragais, 1759. Method of producing improved on by Scheele in 1782.

Tartaric acid, by Scheele in 1768.

Chlorine; produced by Scheele in 1744, and first known as "Dephlogisticated muriatic acid."

Glycerin; discovered by Scheele in 1779 while preparing lead plaster. He called it "The sweet principle of oils."

Lactic acid; Scheele, 1780.

Citric acid; Scheele, 1784.

Gallic acid; Scheele, 1785.

Tannic acid; Berzelius.

Bismuth subnitrate; first used in medicine by L. Odier in 1786.

Sodium carbonate; first recognized as such by Duhamel, 1736.

Thymol; introduced about the middle of the eighteenth century.

OF THE MORE IMPORTANT DISCOVERIES OF THE NINETEENTH CENTURY WE HAVE:

Morphine; discovered in 1804 by Sertürner, an apothecary's assistant, at Paderborn, Germany.

Potassium, sodium, boron, in 1807, and calcium in 1808; by Sir Humphrey Davy, by means of electrolysis.

Iodine; in 1811 by Courtois.

Naphthalin; by Garden in 1816.

Hydrogen dioxide; by Thenard in 1818.

Strychnine; by Pelletier and Caventou in 1818

Veratrine; by Meiszner in 1818.

Brucine; by Pelletier and Caventou in 1819.

Quinine, cinchonine, colchicine; by Pelletier and Caventou in 1820.

Caffeine; Runge, 1820, and independently also by Robiquet in 1821, and by Pelletier and Caventou.

Potassium Iodide; introduced into medicine by D. Coindet in 1821.

Potassium bromide; by Balard in 1826.

Bromine; discovered by Balard in Montpelier, France, in 1826.

Iodoform; produced by Serullus in 1822, but not introduced into the materia medica until about 1837.

Santonin; discovered in 1830, independently of each other, by two German apothecaries, Kahler in Düsseldorf, and Alms in Mecklenburg.

Atropine; isolated by Meins in 1831.

Codeine; by Robiquet in 1832.

Phenol; discovered by Runge, in coal tar, in 1834.

Salicylic acid; in 1839, Kolbe, in 1873, introduced it as an antiseptic.

Chloral hydrate; discovered in 1832 by Liebig. Was introduced as a medicine by Liebreich in 1869.

Chloroform; discovered by Liebig, and also by Soubeiran in 1831. Introduced into medicine by Simpson in 1847.

Theobromine; isolated from the seeds of Theobroma cacao in 1841 by Woskresensky.

Collodion; introduced in 1853 by Maynard and Bigelow.

Physostigmine; by Jobst and Hesse in 1864.

Resorcin; by Hlasiwetz in 1864.

Cocaine; isolated by Gædeke in 1864. Introduced into medicine in 1884 by Koller.

Formaldehyde; discovered by A. W. Hoffman in 1867.

Pilocarpine; Gerrard and Hardy in 1875.

Sodium salicylate; made synthetically by Kolb's process in 1875.

Napthol; 1881.

Apomorphine hydrochloride; used in 1882. The free base had been discovered in 1869 by Matthiessen and Wright.

Antipyrine; discovered by Knorr in 1884.

Acetanilid; discovered by Gerhard, has been in use since 1886.

Phenacetin, 1887. Saccharin, 1887. Sulfonal, discovered by Baumann in 1883. Trional; 1893.

## Figures and Tables

**Fig. 1. f1:**
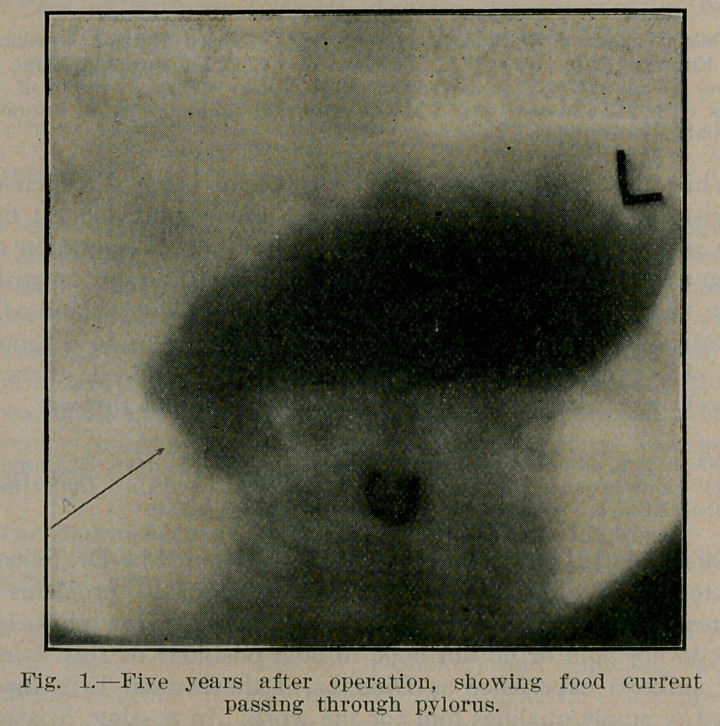


**Fig. 2. f2:**
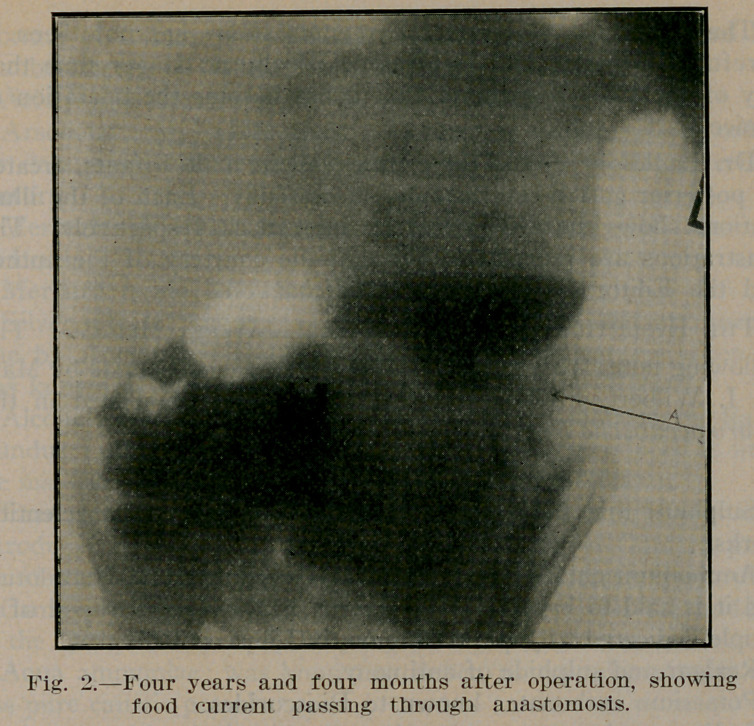


**Fig. 3. f3:**